# The Shear Stress of Host Cell Invasion: Exploring the Role of Biomolecular Complexes

**DOI:** 10.1371/journal.ppat.1004539

**Published:** 2015-01-28

**Authors:** Michelle L. Tonkin, Martin J. Boulanger

**Affiliations:** Department of Biochemistry and Microbiology, University of Victoria, Victoria, British Columbia, Canada; University of Wisconsin Medical School, UNITED STATES

Biological fluids, such as blood and mucosal secretions, continuously flow within the human body and form prominent barriers to pathogen colonization and invasion of host cells [1, 2]. Consequently, pathogens have evolved sophisticated molecular strategies to overcome the mechanical shear forces associated with resisting the flow of biological fluids; in particular, membrane anchored biomolecular complexes enable controlled deceleration, tight adhesion and, in the case of intracellular pathogens, penetration through the host cell membrane (Fig. 1A) [3–7]. The architecture of these biomolecular complexes have been the subject of numerous structural and biophysical investigations, which have yielded high resolution molecular blueprints of the host pathogen interface. However, our understanding of precisely how these biomolecular complexes function is somewhat limited by the technical challenges associated with measuring or modelling the effects of shear flow and related forces on protein complexes in the context of biological membranes [8]; recent studies have revealed the development and application of advanced technologies, such as optical tweezers, for studying the roles that mechanical forces play during pathogen attachment, but the stresses associated with subsequent membrane penetration events remain elusive [3, 7, 9]. To highlight the potential for correlating structural and biophysical data with the extent of dynamic shear stress experienced during diverse host cell invasion processes, we review here several structurally characterized invasion complexes from protozoan, bacterial, and viral pathogens.

**Figure 1:**
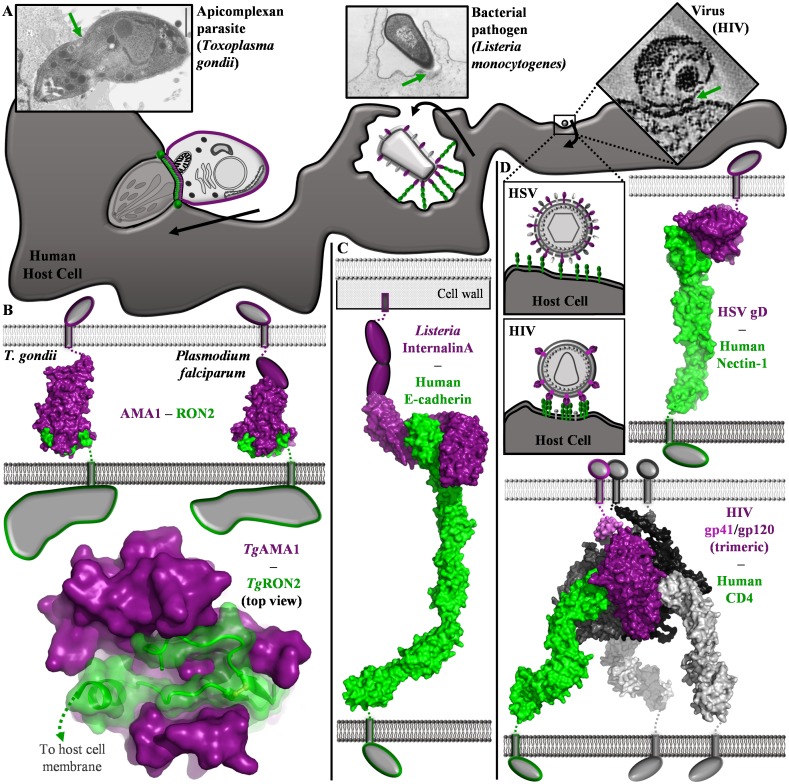
Comparison of structurally characterized invasion mechanisms employed by protozoan, bacterial and viral pathogens. (A) Schematic of invasion mechanisms utilized by *T. gondii*, *L. monocytogenes*, and two enveloped viruses. Electron micrographs: *T. gondii* (reprinted from [19, 40] with permission from Macmillan Publishers Ltd and Elsevier, respectively), *L. monocytogenes* (originally published in [41], reproduced under CC-BY-SA-3.0-us), HIV (reprinted from [42] with permission from Elsevier). Black arrows indicate movement by the pathogen (left) or the host cell (middle), or membrane fusion (right). Green arrows indicate the parasite-host cell interface. (B-D) Thin rectangles, single pass transmembrane domain; top membrane (light grey), pathogen plasma membrane; bottom membrane (dark grey), host plasma membrane; intracellular domains shown as grey shapes (B) Top—apicomplexan parasite-derived AMA1 and RON2 complexes generate a tight link between parasite and host cell (Left: *Tg*AMA1 (Domains I-II-III, purple surface) *Tg*RON2 synthetic peptide (green surface), PDB ID 2Y8T; Right: *Pf*AMA1 (Domains I-II, purple surface; Domain III, purple oval)-*Pf*RON2 synthetic peptide (green surface), PDB ID 3ZWZ). Bottom—apical view of the *Tg*AMA1 (solid purple surface)-*Tg*RON2 (green cartoon and semi-transparent surface) complex. (C) *L. monocytogenes* Internalin A (cap, leucine rich repeat, and Ig-like inter-repeat domains shown as purple surface; C-terminal sequence shown as purple ovals) is anchored in the cell wall and grasps E-cadherin (green surface) in an extended pathogen-host cell link (composite of PDB IDs 1O6S and 3Q2V). (D) Top—HSV gD (purple surface) recognizes human nectin-1 (green surface) (PDB ID 3U82). Bottom—HIV gp41 (violet, dark grey surfaces)-gp120 (purple, dark grey surfaces) trimers coordinate human CD4 (green, light grey surfaces) on T cells (composite of PDB IDs 3LQA, 4NCO and 1WIP).

## Apicomplexan Parasites Provide Both Ligand and Receptor to Form a High Affinity Complex Capable of Supporting Active Invasion of Host Cells

Apicomplexan parasites such as *Toxoplasma gondii* (the etiological agent of toxoplasmosis and one of the most successful parasites on the planet) and *Plasmodium falciparum* (the etiological agent of malaria) actively invade host cells using a highly orchestrated process. Initially, parasites engage host cells through a series of reversible attachments that likely benefit from avidity enhanced clustering of ligands and receptors that effectively bring the parasite and host cell into close proximity [4]. A defining feature of the subsequent tight connection between parasite and host cell is a constricted ring of adhesion formed between the parasite and host cell membranes, termed the moving junction (MJ). Using its own motor complex, the parasite actively propels itself through the MJ ring leading to its encapsulation inside a nascent vacuole within the host cell [10]. Intriguingly, this circumferential ring appears to be formed primarily by parasite proteins. Apicomplexans first secrete their own receptor, rhoptry neck protein 2—RON2, into the host cell, which is then engaged by their own surface ligand, apical membrane antigen 1—AMA1 [11–14]. Thus, reminiscent of the self-reliant enteropathogenic *Escherichia coli* intimin-Tir system used to promote colonization of host cell surfaces [15] but unique among characterized eukaryotic invasion mechanisms, the apicomplexans provide both ligand and receptor to promote host cell invasion. The importance of this mechanism to ensuring successful invasion of the host cell is highlighted both by *T. gondii*’s ability to activate compensatory pairs of AMA1 and RON2 paralogs when the primary AMA1-RON2 complex is compromised [16, 17], and by recent vaccine trials showing that the preformed AMA1-RON2 complex provides significantly more protection than immunization with AMA1 or RON2 alone [18].

Visualization of the MJ using electron microscopy [19] and immunofluorescence reveals that *T. gondii* squeezes through the highly constricted MJ ring (Fig. 1A, left) in a process that is likely to result in substantial mechanical shear forces. Based on structural and biophysical studies, a model was proposed where intimate association between AMA1 and RON2 is sufficiently strong to offset the mechanical stresses of the parasite passing through the constricted MJ ring. This model was largely based on the co-crystal structures of AMA1 and a short region near the C-terminus of RON2 from *T. gondii* and *P. falciparum* (Protein Data Bank (PDB) IDs 2Y8T and 3ZWZ) (Fig. 1B, top) [20, 21], which revealed that a portion of the C-terminal ectodomain of RON2 integrates deeply into a hydrophobic groove on the apical surface of AMA1 (Buried Surface Area (BSA) [22] of 3,765 Å^2^ for *T. gondii* and 3,154 Å^2^ for *P. falciparum*) (Fig. 1B, bottom). Intriguingly, the large BSA of the AMA1-RON2 complex is enabled by substantial conformational change in AMA1 and results in one of the most extensive interfaces of a binary ligand-receptor interaction bridging a pathogen and host cell. Furthermore, binding studies have revealed that the AMA1 has a very high affinity for RON2, with a dissociation constant (K_d_) for *Tg*AMA1-*Tg*RON2 of approximately 15 nM, consistent with the extensive interface [20, 21]. While no evidence of higher order assemblies or avidity enhanced interactions have emerged, the electron dense nature of the MJ ring suggests that AMA1-RON2 complexes are clustered in vivo. Additionally, the intracellular C-terminal domain of AMA1 plays a key role in signaling during parasite invasion [16, 23], and there is the possibility that conformational changes in AMA1 [20, 21, 24, 25] or its clustering into the circumferential MJ ring may be responsible for engaging the parasite motor or signalling productive invasion. Ultimately, elucidating a comprehensive functional profile of the important AMA1-RON2 invasion complex will require its characterization in the context of biological membranes.

## A Bacterial Strategy to Induce Receptor Mediated Internalization into Host Cells

In contrast to the protozoan apicomplexans, attachment of bacteria to host cells is predominately mediated by extended fimbria or pili, and interactions with large extracellular matrix proteins such as fibronectin. However, *Listeria monocytogenes*, a highly virulent food-borne bacterium, is a facultative intracellular pathogen that induces its own internalization through specific receptor mediated interactions (Fig. 1A, middle) [26]. Specifically, Internalin (Inl) A is covalently attached to the cell wall of *L. monocytogenes* and extends out with a curved solenoid domain that grasps E-cadherin on the host cell in a calcium dependent interaction (PDB ID 1O6S; BSA of 2,630 Å^2^; K_d_ of 8 μM in the presence of calcium) [27]. Despite the relatively low affinity of the InlA-E-cadherin interaction, a clustering of receptor-ligand complexes is predicted to mediate macroscopic adhesion [27]. Moreover, the transmembrane adherens junction protein E-cadherin displays a modular architecture with five immunoglobulin-like domains extending away from the host cell membrane that likely function as a flexible connector between the pathogen and host cell (Fig. 1A, middle and 1C). While the InlA interaction with E-cadherin facilitates crossing the intestinal wall, which is the first barrier encountered by *L. monocytogenes*, InlB works in conjunction with InlA by binding the Met tyrosine kinase to affect cell signalling and mediate host cell invasion to a limited extent in the intestinal tract and primarily in deeper tissues (PDB ID 2UZX; BSA of 2,848 Å^2^; K_d_ of approximately 25 nM) [28, 29]. Importantly, exploitation of the human E-cadherin and Met dependent adhesion and signalling system with Inls A and B has enabled *L. monocytogenes* to induce its own uptake in a range of nonphagocytic cells [26].

High resolution molecular blueprints of the InlA and InlB complexes have yielded substantial insight into the invasion mechanisms of *L. monocytogenes*. By characterizing these proteins in a membrane environment, it will be intriguing to determine precisely how these modular receptors (e.g., E-cadherin) and ligands (e.g., InlA) regulate the distance between the *L. monocytogenes* cell wall and host cells membrane, and establish what role this plays in ensuring efficient invasion of host cells. These studies may also help to define how the forces associated with induced phagocytosis compare to those experienced by pathogens invading through alternate mechanisms such as apicomplexan parasites squeezing through the AMA1-RON2 MJ ring, and how low affinity complexes enable macroscopic adhesion.

## Multimeric and Multifunctional Complexes Employed in Viral Host Cell Invasion

The mechanisms employed by viruses to resist shear flow and engage host cells vary substantially, but a common requirement for their genetic material to be successfully internalized by host cells is specific receptor-mediated attachment (Fig. 1A, right). As examples of the diversity in viral adhesion strategies, we will focus here on the multifunctional protein strategy of herpes simplex virus (HSV) and the multimeric protein complex strategy of human immune deficiency virus (HIV).

While the herpes virus, HSV, has multiple surface proteins required for efficient adhesion and subsequent membrane fusion, the major surface adhesin is glycoprotein D (gD), a protein capable of forming higher order homo- and heteromultimeric complexes and coordinating three different classes of receptors: the primary receptor nectin-1 (K_d_ of 17 nM), and secondary receptors herpes virus entry mediator (HVEM) and 3-O-sulfonated heparan sulfate [30, 31]. The complex of HSV gD with two or three of the Ig-like domains of nectin-1 (PDB IDs 3SKU and 3U82, respectively) reveals an average BSA of 1,766 Å^2^ between gD and the N-terminal domain of nectin-1 (Fig. 1D, top) [30, 32]. Intriguingly, the structure of gD in complex with HVEM revealed binding in a region that is distinct from, but overlaps on, the nectin-1 binding site, and also a possible binding site for sulfonated heparan sulfate ligands (PDB ID 1JMA) [33]. From these costructures in combination with the structure of apo gD, it has been speculated that conformational changes in gD induced by receptor binding provide a signal that initiates fusion between the viral and host cell membranes [30, 32, 34].

In contrast, the surface of the AIDS virus, HIV, is dominated by cleavage products of envelope glycoprotein 160 (gp160), which is proteolytically processed into two fragments: membrane bound gp41 and noncovalently associated gp120. Trimers of gp41-gp120 heterodimers are anchored in the viral membrane, and gp120 recognizes CD4 primarily on human T cells. Based on structural studies of gp120 with the terminal immunoglobulin domain of CD4, each gp120-CD4 complex buries 1,950 Å^2^ of surface area, indicating an intimate association consistent with the measured K_d_ of 5 nM [35, 36]. Interestingly, the thermodynamics of the gp120-CD4 interaction suggest that a large conformational reorganization in gp120 is required for coordination of CD4 [36]. Since CD4 is comprised of four immunoglobulin-like domains, the gp41-gp120-CD4 link generates an extended link between the viral and host cell membranes (Fig. 1C, bottom). Not surprisingly, a subsequent, closer interaction between gp120 and a chemokine receptor (e.g. CXCR4 or CCR5) is required to bring the membranes into sufficiently close apposition to enable viral fusion with the host cell. The transmembrane nature of both CD4 and chemokine receptors is exploited by HIV, as binding of gp120 induces signalling events that prepare the host cell for viral entry [37].

Despite the structural and biophysical insights gained into these and other viral invasion strategies, it remains unclear precisely how the affinity, avidity, and conformational changes of these membrane anchored biomolecular complexes regulate the subsequent steps leading to membrane fusion and viral entry.

## Conclusions and Implications for Future Studies

The structural study of biomolecular complexes that drive pathogen internalization into host cells offers targeted strategies for therapeutic development and enhances our basic understanding of molecular pathogenesis. In particular, the molecular assemblies reviewed here have contributed to the development of new drug targets [38, 39] and provided insight into novel strategies to enhance vaccine efficiency against global diseases such as AIDS and malaria [18, 36]. Furthermore, our comparison of just four protein complexes that allow pathogens to resist shear forces prevalent throughout the human body reveals an array of strategies enabled by an exceptionally diverse set of molecular interactions. However, attempting to correlate the high resolution structural data to precise roles in host cell invasion reveals many critical unanswered questions: Is there a direct relationship between the affinity, avidity, or BSA of a host-pathogen receptor-ligand complex and the shear stress that must be overcome to enable invasion? What role does shear flow play in functionally activating ligands and receptors? In situations of pathogen ligand binding sites overlapping the binding site for the native host cell ligand (e.g., HSV gD/nectin-1 versus nectin-1/nectin-1), have high affinity interactions evolved at least partially to overcome competition with the host complexes? In the case of ligand and receptor originating from the pathogen (e.g., apicomplexan AMA1-RON2), are high affinity interactions the result of optimized force resistance since no competition with a native host ligand occurs? Do mechanical forces play a role in how protein-protein complex formation at the host-pathogen interface is related to transmitting forward (i.e., into the host cell) and/or reverse (i.e., into the pathogen) signals that are important for successful invasion events? What roles do conformational change of pathogen ligands (e.g., apicomplexan AMA1, HSV gD, HIV gp120) play in regulating specificity, signal transduction, and immune evasion and do these conformational changes differ in the context of a membrane? Overall, our understanding of the intricate roles of biomolecular complexes linking pathogen and host cell membranes is greatly enhanced by the in vitro high resolution biophysical characterization of the isolated complexes such as those presented here. However, a detailed and comprehensive description of molecular invasion strategies will continue to rely on the development and application of innovative and multidisciplinary approaches to ensure data are interpreted in the context of biologically relevant environments.
